# The effectiveness of creatine treatment for Parkinson’s disease: an updated meta-analysis of randomized controlled trials

**DOI:** 10.1186/s12883-017-0885-3

**Published:** 2017-06-02

**Authors:** Jia-Jie Mo, Lin-Ying Liu, Wei-Bin Peng, Jie Rao, Zhou Liu, Li-Li Cui

**Affiliations:** 1 Guangdong Key Laboratory of Age-Related Cardiac and Cerebral Diseases, Affiliated Hospital of Guangdong Medical University, No.2 Wenming Road, Zhanjiang, Guangdong 524023 People’s Republic of China; 2 Department of Neurology, Affiliated Hospital of Guangdong Medical University, No.2 Wenming Road, Zhanjiang, Guangdong 524023 People’s Republic of China; 3 Department of Neurology, Chenzhou No. 1 People’s Hospital, Chenzhou, People’s Republic of China; 4 Graduate School, Guangzhou Medical University, Guangzhou, City, People’s Republic of China; 5 Department of Pathology, People’s Hospital of Wuhan University, Wuhan City, People’s Republic of China

**Keywords:** Creatine, Meta-analysis, Mitochondrial dysfunction, Parkinson’s disease

## Abstract

**Background:**

The effectiveness of creatine in treating Parkinson’s disease (PD) has not been conclusively determined. Therefore, we performed a meta-analysis to address this issue.

**Methods:**

The Cochrane Central Register of Controlled Trials, PUBMED, EMBASE, and other databases were searched, and outcomes measured by the Total Unified Parkinson’s Disease Rating Scale (UPDRS) and the Schwab & England Scale were analyzed.

**Results:**

Five randomized controlled trials (RCTs) were selected, and 1339 participants were included in the analysis. There were no significant differences between the control and treatment groups in the total, mental, activities of daily living (ADL), or motor UPDRS scores, but an improvement in Schwab & England Scale scores was observed.

**Conclusions:**

Creatine has no observed benefit in PD patients, although more correlated studies are still needed.

## Background

Parkinson’s disease (PD) is a common, progressive, neurodegenerative condition that causes both motor (stiffness, slowness, rest tremor, and poor postural reflexes) and non-motor symptoms (abnormalities in mood, cognition, sleep, and autonomic function). The incidence of PD ranges from 8 to 18 per 100,000 person-years [1], and thus identifying effective drugs that can slow the progression of PD is critical. PD is not purely a disorder of the basal ganglia; it also has systemic causes. For instance, a variety of mechanisms including oxidative stress, excitotoxicity, apoptosis, and mitochondrial dysfunction can all contribute to PD [2, 3]. Additionally, disease pathogenesis results not only from the loss of dopaminergic neurons in the substantia nigra pars compacta but also from deposits of α-synuclein in the peripheral nervous system and deterioration of small nerve fibers [4]. Existing drugs such as levodopa, dopamine agonists, monoamine oxidase inhibitors (MAO-B inhibitors), and catechol-O-methyltransferase inhibitors (COMT inhibitors) are not completely effective in PD patients. Therefore, neuroprotective agents such as creatine have increasingly been considered for their potential efficacy [5–9].

Creatine is a natural compound that plays an important role in cellular energy homeostasis. It can be converted to phosphocreatine [10], an energy intermediate that can then transfer a phosphoryl group to synthesize mitochondrial ATP. Creatine exhibits anti-apoptotic, anti-excitotoxic and direct antioxidative properties [5]. Previous studies have reported that homocysteine accumulation may eventually lead to peripheral nerve damage [11, 12] and that creatine exhibits neuroprotective and antioxidant properties [13] by reducing homocysteine levels [14]. Therefore, creatine may be effective in treating neurodegenerative diseases [15–17].

Previous studies have reported conflicting results regarding the effect of creatine as a treatment for PD, and it remains unclear whether creatine treatment can improve clinical outcomes when compared with a placebo [18–22]. Therefore, this meta-analysis was conducted to investigate the symptomatic efficacy of creatine versus placebo, analyzing outcomes assessed by the Unified Parkinson’s Disease Rating Scale (UPDRS) and the Schwab & England Activities of Daily Living Scale.

## Methods

### Literature search

Two authors independently conducted a systematic literature search of multiple databases, including the Cochrane Central Register of Controlled Trials, PUBMED, EMBASE, and other sources, for studies published through October 2016. We also searched internet-based clinical trial registries, such as the clinical trial registry (Clinicaltrials.gov) and the OpenGrey database (a system for information on the gray literature in Europe). The following keywords and MeSH terms were used: “creatine”, “Parkinson’s disease”, and “randomized controlled trials”.

### Study selection

Trials were included in our meta-analysis if they met all the following criteria: (1) they were randomized, double-blinded, controlled trials (RCTs); (2) the recruited patients met the UK Parkinson’s Disease Society Brain Bank Clinical Diagnostic Criteria and their PD-MCI diagnosis was based on the Criteria for the Diagnosis of PD-MCI [23] formulated by the Movement Disorder Society (MDS) of the United States; (3) the intervention therapies included creatine; and (4) the efficacy was assessed by the UPDRS or the Schwab & England Scale. Two of the authors independently applied these selection criteria to screen studies for eligibility.

### Data extraction

The data were collected in two steps. First, the title and abstract of each study were recorded, and studies that were either clearly irrelevant or were duplicates were removed. Second, the full text of articles that passed the initial screening was retrieved, and the eligibility of these studies for this meta-analysis was determined after collecting the following information: last name of the first author, year of publication, study design details, methodological quality (assessed using domain-based evaluations [Cochrane Handbook 8.3.1]), patient characteristics (including gender, age, disease duration, and baseline disease severity), sample size, treatment dose, duration of treatment, and study outcomes (changes in Mental UPDRS, activities of daily living (ADL) UPDRS, Motor UPDRS, Total UPDRS or Schwab & England scores). Multiple authors were involved in the screening, and any disagreements regarding eligibility were resolved through careful discussion.

### Quality assessment

Two authors independently assessed the validity of each study by determining the risk of bias according to the Cochrane Handbook for Systematic Reviews of Interventions (Version 5.1.0) [24]. Any disagreements in the authors’ assessments were resolved though discussion. Specifically, the studies were evaluated considering the following criteria: random sequence generation, allocation sequence concealment, blinding of outcome assessment, incomplete outcome data and other latent threats to validity. The risk of bias was categorized as “high”, “unclear” or “low”.

### Level of evidence

We determined the quality of evidence in each study using the Grades of Recommendation, Assessment, Development and Evaluation (GRADE) approach. GRADE profiler 3.6 software (Cochrane Collaboration, http://tech.cochrane.org/revman/other-resources/gradepro/download) was used to create an evidence profile, and each study was characterized as follows: (1) high quality (A), indicating that further research was extremely unlikely to change our confidence in the estimate of effect; (2) moderate quality (B), indicating that further research was likely to have an important impact on our effect estimate and could change the estimate; (3) low quality (C), indicating that further research was extremely likely to have an important impact on our effect estimate and could change the estimate; and (4) very low quality (or D), indicating that we were extremely uncertain about the estimate of effect.

### Statistical analysis

The data analyses were performed using Review Manager Software (Version 5.3). The mean changes from baseline in the UPDRS scores and the Schwab & England Scale scores were treated as continuous variables, and the weighted mean differences (WMDs) with 95% confidence intervals (CIs) were calculated as the difference between the effects of creatine and placebo treatment. Heterogeneity, assessed by the standard Cochran Q and I-squared measures, was determined by *P* < 0.10 or I-squared >50% in each study. When there was homogeneity between trials, a fixed-effect model approach was used to combine the trial outcomes [25], while a random-effects model was used in cases of heterogeneity [26].

## Results

### Search results and study characteristics

**Fig. 1:**
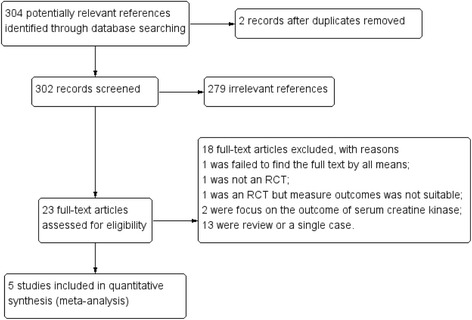
Assessing the risk of bias of the included studies. **a** A summary of the authors’ evaluation of the risk of bias criteria for each included study; (**b**) A graph of the authors’ evaluation of each risk of bias criterion presented as percentages across all included studies Flow chart with inclusion criteria to select studies for this meta-analysis

**Fig. 2:**
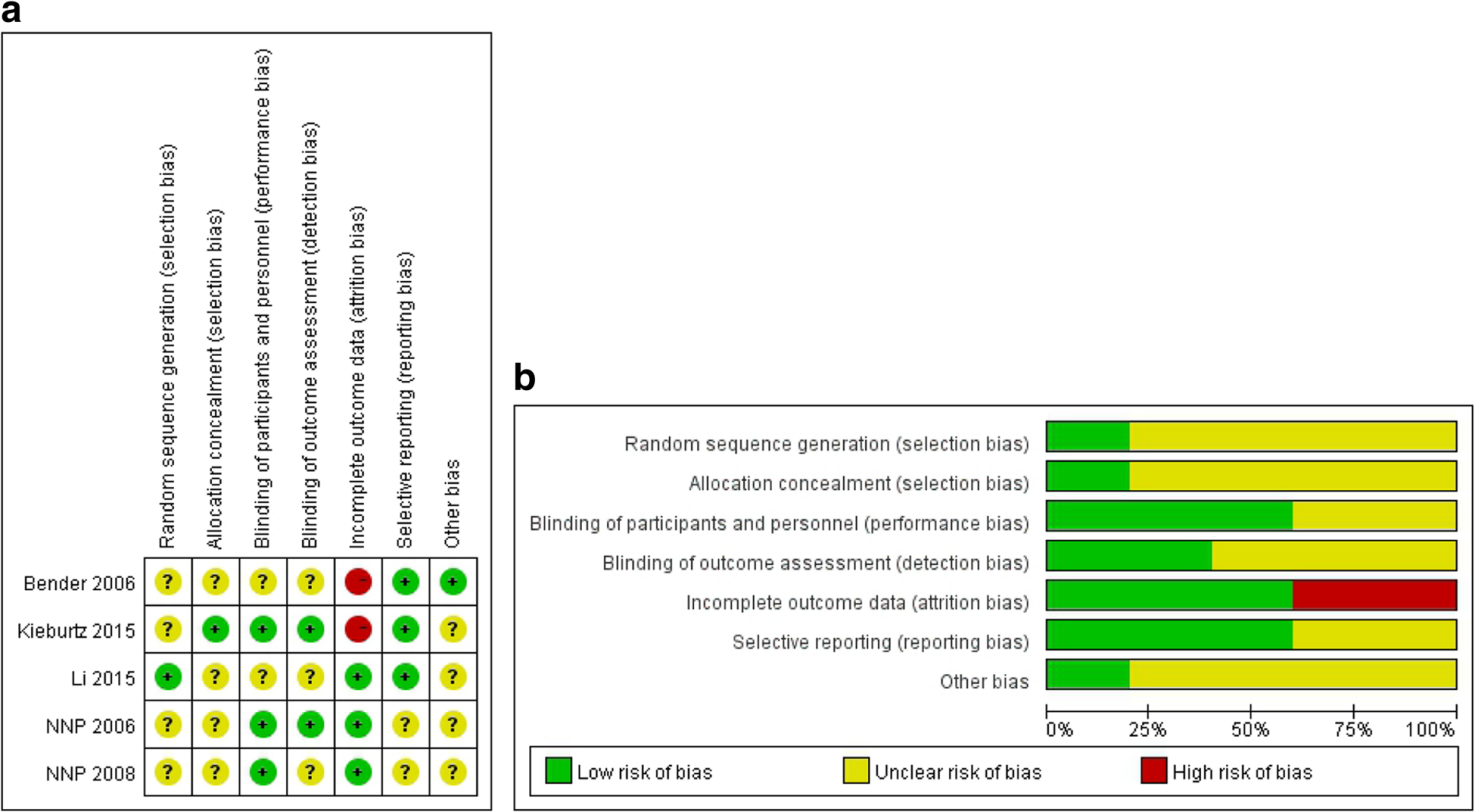
Assessing the risk of bias of the included studies. (**a**) A summary of the authors’ evaluation of the risk of bias criteria for each included study; (**b**) A graph of the authors’ evaluation of each risk of bias criterion presented as percentages across all included studies

**Fig. 3:**
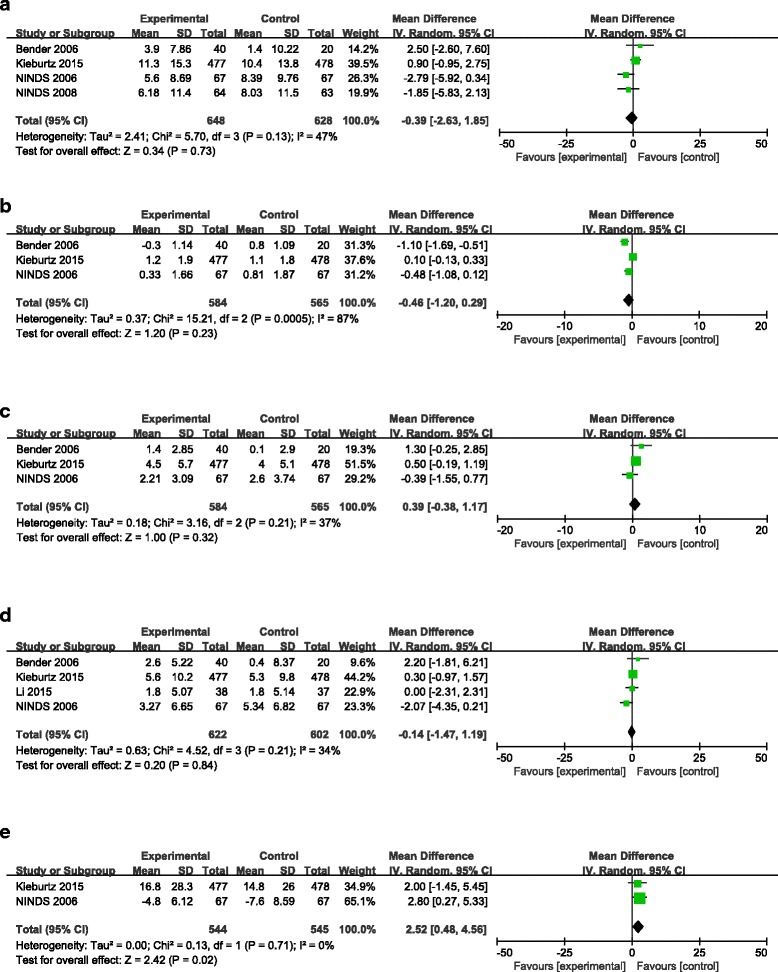
The effect of creatine treatment versus placebo. (**a**) Change in Total UPDRS scores from baseline; (**b**) Change in UPDRS Mental scores; (**c**) Change in UPDRS ADL scores; (**d**) Change in UPDRS Motor scores; (**e**) Change in Schwab & England Scale scores

The search strategy initially identified 302 articles published prior to October 2016. Twenty-three of these articles were selected, and the full papers were analyzed to assess the efficacy and safety of creatine treatment in PD patients. Of these 23 papers, 17 were excluded and 1 was incomplete, although it may have satisfied the inclusion criteria [27], leaving 5 studies available for the final analysis [[18–22]; Fig. 1]. The main characteristics of the included studies and patient populations are summarized in Table 1 and Table 2, respectively. In one study, creatine was administered at a loading dose of 20 g/d for 6 days, followed by 2 g/d for 6 months and finally 4 g/d for 2 years [21]. Another study combined creatine (10 g/d) with coenzyme Q10 (300 mg/d) [18]. Patients in the other 3 studies all received 10 g/d of creatine [19, 20, 22]. Disease progression was primarily measured using the UPDRS. Three of the five studies assessed disease progression using the Total, Mental, ADL, and Motor UPDRS scores. One study used the Total UPDRS scores only [20], and one study used the UPDRS Motor scores only [18]. Two studies provided data based on the Schwab & England scale [19, 22]. The assessment of risk of bias among included studies was shown in the Fig. 2. The meta-analyses of the studied outcomes were shown in the Fig. 3.

**Table 1:** Characteristics of the studies included in the meta-analysis

Author	Year	Method	Country	Participants		Intervention(s)	Outcomes
				Control	Placebo		
NINDS [22]	2006	RCT	USA	67	67	Creatine 10 g/d and Minocycline 200 mg/d	Total UPDRS scores; UPDRS I/II/III
Bender [21]	2006	RCT	Germany	40	20	Creatine 4 g/d	Total UPDRS scores; UPDRS I/II/III
NINDS [20]	2008	RCT	USA	64	63	Creatine 10 g/d	Total UPDRS scores;
Kieburtz [19]	2015	RCT	Canada USA	477	478	Creatine 10 g/d	Total UPDRS scores; UPDRS I/II/III Schwab & England Scale
Li [18]	2015	RCT	China	38	37	Creatine 10 g/d and Coenzyme Q10 300 mg/d	UPDRS III Schwab & England Scale

*RCT* Randomized controlled trial, *UPDRS* Unified Parkinson’s Disease Rating Scale

**Table 2:** Baseline characteristics of the patient populations

Author	Intervention(s)	Total UPDRS score	UPDRS Mental score	UPDRS ADL score	UPDRS Motor score	Hoehn & Yahr scale	Schwab & England scale	Follow-up Duration (months)	Disease Duration (years)
NINDS 2006 [22]	Creatine 10 g/d	23.9(9.07)	1.13(1.29)	6.33(3.07)	16.4(6.77)	1.43(0.5)	92.7(5.25)	12	Within 5 yrs
	Placebo	22.8(9.63)	1.13(1.19)	6.03(3.45)	15.6(7.01)	1.46(0.5)	94.2(4.81)		
Bender 2006 [21]	Creatine 4 g/d	27.4(11.7)	2.2(1.9)	8.1(4.6)	16.3(7.0)			24	Above 2 yrs
	Placebo	27.4(17)	1.6(1.5)	7.8(4.8)	17.4(11)				
NINDS 2008 [20]	Creatine 10 g/d	23.9(9.07)	1.13(1.29)	6.33(3.07)	16.4(6.77)	1.43(0.5)	92.7(5.25)	18	Within 5 yrs
	Placebo	22.8(9.63)	1.13(1.19)	6.03(3.45)	15.6(7.01)	1.46(0.5)	94.2(4.81)		
Kieburtz 2015 [19]	Creatine 10 g/d	26.5(11.7)	1.3(1.4)	7.3(4.1)	17.9(8.6)		90.9(6.6)	60	Within 5 yrs
	Placebo	25.9(11)	1.3(1.4)	7.0(3.8)	17.6(8.1)		91.4(6.3)		
Li 2015 [18]	Creatine 10 g/d				17.5(7.8)			18	Approximately 7.7 yrs
	Placebo				18.8(7.4)				

Values: weighted mean difference (standard deviation)

### The effect of Creatine vs. placebo treatment on Total UPDRS scores

Four of the trials included in this analysis reported data on the effects of creatine vs. placebo on Total UPDRS scores [19–22]. As shown in Table 3, the pooled WMD of the Total UPDRS scores at the end of the follow-up period in patients who received creatine compared to those who received placebo was −0.39 (95% CI = [−2.63, 1.85], *P* = 0.73), with no significant heterogeneity (*P* = 0.13, I^2^ = 47%); this finding indicating that creatine treatment did not affect PD patients’ Total UPDRS scores differently than placebo.

**Table 3:** Efficacy results

Outcome	Studies	Participants	Heterogeneity	WMD (95% CI]
Total UPDRS score	4	1276	Chi^2^ = 5.70, df = 3 (*P* = 0.13); I^2^ = 47%	−0.39 [−2.63, 1.85]
UPDRS Mental score	2	194	Chi^2^ = 2.08, df = 1 (*P* = 0.15); I^2^ = 52%	−0.79 [−1.40, −0.18]
UPDRS ADL score	3	1149	Chi^2^ = 3.16, df = 2 (*P* = 0.21); I^2^ = 37%	0.39 [−0.38, 1.17]
UPDRS Motor score	4	1224	Chi^2^ = 4.52, df = 3 (*P* = 0.21); I^2^ = 34%	−0.14 [−1.47, 1.19]
Schwab & England Scale score	2	1089	Chi^2^ = 0.13, df = 1 (*P* = 0.71); I^2^ = 0%	2.52 [0.48, 4.56]

WMD: weighted mean difference; 95% CI: 95% confidence interval

### The effect of Creatine vs. placebo treatment on UPDRS mental scores

In three of the five studies [19, 21, 22], UPDRS Mental scores were also used to assess the effects of creatine vs. a placebo in patients with PD (Table 3). The pooled WMD for UPDRS Mental scores at the end of the follow-up period in patients who received creatine compared to those who received placebo was −0.46 (95% CI = [−1.20, 0.29], *P* = 0.23) and was associated with statistically significant heterogeneity (*P* = 0.0005, I^2^ = 87%).

### The effect of Creatine vs. placebo treatment on UPDRS ADL scores

Data on the effect of creatine vs. placebo treatment on UPDRS ADL scores were reported in threestudies [19, 21, 22]. As described in Table 3, the pooled WMD for the scores at the end of the follow-up period in patients who received creatine compared to those who received placebo was 0.39 (95% CI = [−0.38, 1.17], *P* = 0.32), with non-significant heterogeneity (*P* = 0.21, I^2^ = 37%), indicating that creatine treatment and placebo had similar effects on PD patients’ UPDRS ADL scores.

### The effect of Creatine vs. placebo treatment on UPDRS motor scores

Four of the studies [18, 19, 21, 22] explored the effects of creatine vs. placebo treatment on UPDRS Motor scores (Table 3) and found that the pooled WMD for UPDRS Motor scores at the end of the follow-up period in patients who received creatine compared to those who received placebo was −0.14 (95% CI = [−1.47, 1.19], *P* = 0.84), with non-significant heterogeneity (*P* = 0.21, I^2^ = 34%). This result suggested that creatine treatment did not have a greater effect than placebo on PD patients.

### The effect of Creatine vs. placebo treatment on Schwab & England Scale scores

Two of the studies examined here [19, 22] explored the effects of creatine vs. placebo treatment on Schwab & England Scale scores (Table 3). The pooled WMD of Schwab & England Scale scores at the end of the follow-up period in patients who received creatine compared to those who received placebo was 2.52 (95% CI = [0.48, 4.56], *P* = 0.02), and the results showed statistically significant heterogeneity (*P* = 0.71, I^2^ = 0%). These findings indicated that PD patients who used creatine may have had a higher Schwab & England Scale score than those who received the placebo.

### Risk of bias in the included studies

The risk of bias of each parameter in each study was also assessed (Fig. 2). One study [19] reported a high drop-out rate (greater than 20%), and this attrition bias posed a high risk. Overall, no studies were deemed completely free of risk of bias. We also assessed the quality of evidence in each study using the GRADE scale (Table 4).

**Table 4:**
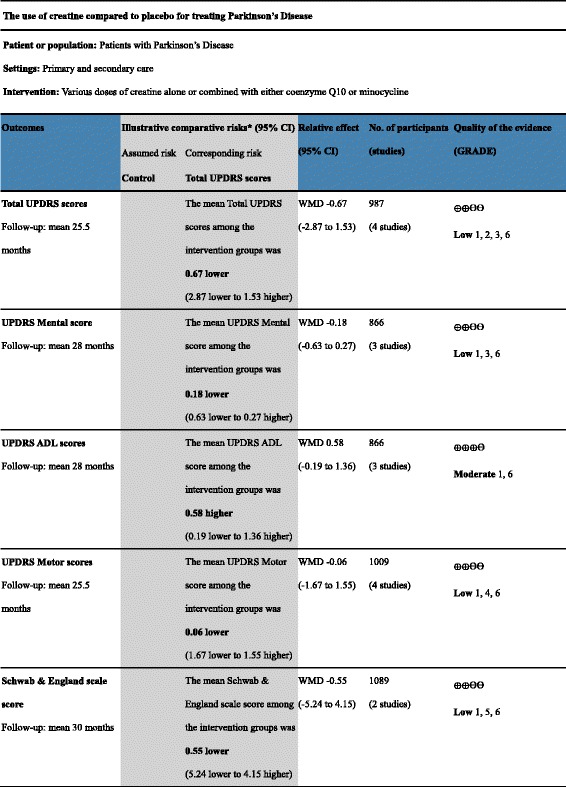
GRADE evidence profile for all included studies

*Basis for the assumed risk (e.g., the median control group risk across studies) is provided in the footnotes. The corresponding risk (and its 95% confidence interval) is based on the assumed risk in the comparison group and the relative effect of the intervention (and its 95% CI). CI: Confidence interval

GRADE Working Group categories for quality of evidence

High quality: Further research is very unlikely to change our confidence in the estimate of effect.

Moderate quality: Further research is likely to have an important impact on our confidence in the estimate of effect and may change the estimate

Low quality: Further research is very likely to have an important impact on our confidence in the estimate of effect and is likely to change the estimate

Very low quality: We are very uncertain about the estimate of effect

1 Evidence includes lack of allocation concealment and random sequence generation

2 A moderate variation in baseline variables between trials

3 The *p* value for heterogeneity was greater than 0.05, and I^2^ was 50% in the comparison of four studies; however, the quality of the evidence was not downgraded because it was considered low risk

4 Two trials [18, 22] used creatine combined with CoQ10 or minocycline as a co-intervention. The intervention was not confined to a single variation, which may induce an indirect influence

5 The *p* value for heterogeneity was less than 0.05, and I^2^ was 79% in this comparison with 2 studies. The quality of the evidence was downgraded for inconsistency

6 The rate of drop-outs in the trial [19] was high and greater than 20% (397/894 in the creatine group and 289/867 in the placebo group)

## Discussion

In our meta-analysis of five previously published papers, we assessed the effectiveness of creatine treatment versus placebo in PD patients. We found no association between creatine treatment and a decreased Total, Mental, ADL, or Motor UPDRS score, but we did note a greater improvement in Schwab & England Scale scores with use of creatine compared to placebo; however, this last test was included in 2 studies only. Both the UPDRS ADL and the Schwab & England Scale assess whether patients show improvement in ADL; however, this analysis indicated conflicting outcomes. Therefore, we believe that not enough evidence exists to support the theory that creatine can enhance ADL.

Conventional medications have been ineffective in curing PD to date, and creatine treatment has emerged as a potential method for slowing the progression of PD. Correlated basic studies have suggested that creatine does have a positive effect on PD patients, and these initial results led to the initiation of multiple clinical trials [18–22]. However, consensus has not been reached on whether this treatment is effective, which is what prompted this meta-analysis. Our findings may provide clinicians with an alternative approach to treating PD. Although another meta-analysis on this subject was performed by Xiao et al. (2014) [28] and involved two RCTs with a total of 194 patients, the findings were unreliable because of the high risk of bias, the small sample sizes and the short duration of the eligible trials. The efficacy of creatine treatment for PD requires data from clinical trials before any conclusions can be reached. The systematic review conducted here assessed the efficacy and safety of creatine as a primary or adjuvant treatment for PD. We included five RCTs [18–22] that had a total of 1339 patients and compared creatine treatment with the administration of a placebo. To evaluate the PD patients’ prognosis, we assessed whether their UPDRS scores or Schwab & England Scale scores had changed after treatment. We addressed the limitations of the previous meta-analysis by performing a comprehensive and extensive literature search that evaluated RCTs using appropriate criteria, including a qualitative analysis of RCTs and the use of the GRADE approach to determine the quality of evidence. The quality of the studies was considered only moderate or low based on the following parameters: [1] the included trials were reported as randomized, double-blind trials but did not provide additional details; [2] moderate variation in baseline variables was observed among the studies; [3] some comparisons exhibited heterogeneity; and [4] creatine was used in combination with CoQ10 or minocycline [18, 22]. The RCTs were generally of high quality, although we did include RCTs that involved diverse risks. Overall, the methodological quality of the 5 enrolled studies was considered good with respect to the most common and relevant biases.

Because the high number of patients enrolled in the study by Kieburtz et al. [19] accounted for a large proportion of our overall analytical sample (955/1339; 71.3%), a sensitivity analysis was performed and found no evidence of significant heterogeneity. We found that this single study did influence the overall outcome, resulting in a significant decline in UPDRS Mental scores. Although a sensitivity analysis was performed and found no evidence of significant heterogeneity. This influence might be due to the large sample size, the longer follow-up duration or the high rate of drop-outs that had to be excluded. These and other confounding factors, such as follow-up duration, disease duration, and the baseline disease severity of the patients in each study, are fully described in Table 2. The baseline characteristics of the patient population were similar when initially assessed. Regarding follow-up duration, only the study by Kieburtz et al. [19] lasted longer than 24 months. This parameter, together with the high rate of drop-outs in that same study, may account for the heterogeneity observed when examining the UPDRS Mental scores. Additionally, the disease duration in the study by Li et al. [18] was longer than the duration reported in the other 4 studies, and this discrepancy may or may not have contributed to the significant heterogeneity observed.

There are some limitations to this meta-analysis. First, although we included three more studies than Xiao et al. (2014), the per-study and overall sample size remained small. Second, the disease outcomes were primarily assessed by the UPDRS and Schwab & England Scale, which may not cover other potential treatment benefits for patients with psychiatric and cognitive disorders. Finally, these studies lacked long-term assessments of key indicators, such as 5- or 10-year patient survival rates. Some of the trials were quite heterogeneous, with several reasonable explanations for this finding: [1] the trials used different doses of creatine; [2] two of the trials [18, 22] used CoQ10 or minocycline as a co-intervention; [3] all of the trials were reported as RCTs but did not provide additional details; [4] the intervention was not confined to a single variant, which may have had indirect effects; and [5] one study [19] reported a high drop-out rate (greater than 20%.)

Based on this meta-analysis, we believe that creatine treatment is ineffective and that it has limited prospects as a drug of choice for PD. The limited efficacy of creatine in treating PD can be explained by several factors. First, the cellular transport of this substance is constrained [29]. In a previous study, after patients were treated with creatine (3.4 g/d) for 4 weeks, brain creatine levels were only slightly elevated [30]. Another factor is the insufficient dose administered. Compared to rodents, which were given 4 g of creatine per day in their food (133 g/kg), humans who received the highest dose of 10 g of creatine per day (0.15 g/kg for a 65-kg adult) still did not experience the same positive effects [21]. Moreover, in a study that involved mitochondrial dysfunction, creatine was found to be significantly correlated with depression [31]. One study we included in this analysis found that patients treated with creatine had a significantly better score on the Mental UPDRS (*P* = 0.046) [21], while another found that creatine combined with Coenzyme Q10 could delay the decline in cognitive function of patients with PD, as assessed by the Montreal Cognitive Assessment (MoCA) [18]. Thus, in addition to focusing on the efficacy of creatine in relieving the motor symptoms of PD, we should also examine its effect on the psychiatric and cognitive symptoms.

Although the effectiveness of creatine in improving mitochondrial function has previously been demonstrated, it showed no effect on PD patients in this analysis. Accordingly, we hypothesize that mitochondrial dysfunction may play an indirect role in the development of PD, which may be susceptible to many other underlying but yet unknown factors. Therefore, the use of other neuroprotective agents to treat PD should still be investigated.

## Conclusion

Creatine was not found to be effective in treating patients with PD, although more evidence is needed to determine whether it can improve their ADL. In addition, future studies that determine whether the drug might be useful in treating psychiatric and cognitive disorders should be conducted.

## Abbreviations

95%CI: 95% confidence interval
ADL: Activities of daily living
PD: Parkinson’s disease
RCT: Randomized controlled trial
UPDRS: Unified Parkinson’s Disease Rating Scale
WMD: Weighted mean differences
